# Dual-incision minimally invasive fasciotomy of the anterior and peroneal compartments for chronic exertional compartment syndrome of the lower leg

**DOI:** 10.1038/s41598-020-75268-2

**Published:** 2020-10-22

**Authors:** Christoph Grechenig, Epaminondas Markos Valsamis, Amir Koutp, Gloria Hohenberger, Theresa di Vora, Peter Grechenig

**Affiliations:** 1 Department of Ophthalmology, University of Vienna, Währinger Gürtel 18-20, 1090 Vienna, Austria; 2 Nuffield Orthopaedic Centre, Oxford University Hospitals NHS Foundation Trust, Oxford, OX3 7LD UK; 3 Medical University of Graz, Auenbruggerplatz 5, 8036 Graz, Austria; 4 Department of Orthopaedics and Trauma Surgery, Medical University of Graz, Auenbruggerplatz 5, 8036 Graz, Austria; 5 Division of Macroscopic and Clinical Anatomy, Medical University of Graz, Harrachgasse 21, 8010 Graz, Austria

**Keywords:** Musculoskeletal system, Trauma

## Abstract

To evaluate the risk of iatrogenic injury when using a dual-incision minimally invasive technique to decompress the anterior and peroneal compartments of the lower leg. Forty lower extremities from 20 adult cadavers, embalmed with Thiel’s method, were subject to fasciotomy of the anterior and peroneal compartment using a dual-incision minimally invasive fasciotomy. The first incision was made 12 cm proximal to the lateral malleolus to identify and protect the superficial peroneal nerve (SPN). The second incision was made at the mid-point of the Fibula (half-way between the fibular head and the lateral malleolus). Release of the anterior and peroneal compartments was successful in all specimens. Two nerve injuries of the superficial peroneal nerve were reported. More precisely, in these cases the medial dorsal cutaneous nerve got injured during the fascial opening of the extensor compartment. Two incision minimally invasive fasciotomy to decompress the anterior and peroneal compartments of the lower leg appears to be safe with regard to the results of this study.

## Introduction

Surgical decompression of the lower leg for acute compartment syndrome is the only reliable way to prevent the late sequelae of ischemic contracture if it is done in time. Furthermore, the longer decompression is delayed, the greater will be the degree of functional loss^1^.

A rare but accepted clinical diagnosis in runners and military recruits is a chronic exertional compartment syndrome (CECS)^2^. The treatment of choice, after failed conservative management, is surgical fasciotomy^3^.

The literature varies widely on postoperative outcomes for fasciotomies in CECS but are acceptable. Success rates for these procedures are between 52 and 100%^4–6^. Minimally invasive techniques are gaining popularity. This is because they have shown low recurrence rates and allow a faster return to sports activities^7,8^. The literature heavily focused on techniques for decompression of the anterior compartment, as it is assumed that the anterior compartment of the leg is most frequently affected^9,10^.

The risk of neurovascular injury is a major surgical problem in the application of minimally invasive fasciotomies. The superficial peroneal nerve (SPN) is anatomically closely related to the anterior and peroneal compartments, with the rate of reported iatrogenic SPN injuries varying between 0–8%^7,10^. This discrepancy reflects both the differences in surgical techniques as well as the small number of cases studied, particularly in cadaveric studies^10^.

Hutchinson et al. reported a concerningly high risk (67%) for iatrogenic neurovascular damage with minimally invasive fasciotomy without using an endoscope, however the study is limited due to a small number of specimens (n = 6)^11^.

Pacha et al.^12^ evaluated the perforation point of the SPN of the superficial crural fascia. The level at which the SPN pierces the superficial fascia in the lower leg and penetrates into the subcutaneous tissue was on average at 116.8 mm proximal to the lateral malleolus; i.e., in 95% of cases the SPN emergence was between 166 and 66 mm proximal to the lateral malleolus^12^.

The aim of this study was to evaluate the safety of a two incision minimally invasive fasciotomy with a secure technique to prevent iatrogenic injuries without the use of endoscopy for lower leg CECS when decompressing the anterior and peroneal compartments.

## Materials and methods

40 lower extremities from 20 adult human cadavers embalmed with Thiel’s method were used for this study^13^. Cadavers with macroscopically obvious pathology of the lower limb were not used. The cadavers were donated to the Medical University of Graz.

Each cadaver was placed in a supine position on a dissection table with support devices beneath both knees. Fasciotomy of the anterior and peroneal compartments was undertaken in all cadaveric specimens. The procedure was first demonstrated by a highly experienced trauma surgeon using muscle specimen and then demonstrated on two lower extremities, which were assisted by students who worked as anatomical instructors at the Anatomy of Graz. Subsequently, the students performed the fasciotomies, where one of them prepared and the other assisted. All fasciotomies were performed under the supervision of the trauma surgeon.

After the releases were completed, careful anatomical dissection was carried out to identify incomplete division of the fascia, muscle injury (division of muscle fibres), neurovascular injury and the anatomical relationship of key neurovascular structures to the incisions.

In addition, the distances of the respective fascial openings from the anterior and peroneal compartments at the most proximal and distal points to each other and to the tibial crease were measured.

### Fasciotomy of the anterior and peroneal compartments

#### First incision

The basic-reference point (12 cm proximal to the lateral malleolus), as described in the technique by Pacha et al.^12^, was identified and therefore a two cm horizontal skin incision was performed. The subcutaneous layer was carefully dissected using dissecting scissors in each specimen to avoid distortion of neural structures. The SPN which runs dorsal to the septum was determined and care was taken not to manipulate their course. The anterior and peroneal compartments were opened separately through minimal invasive, longitudinal fascial incisions using long surgical scissors.

**Figure 1:**
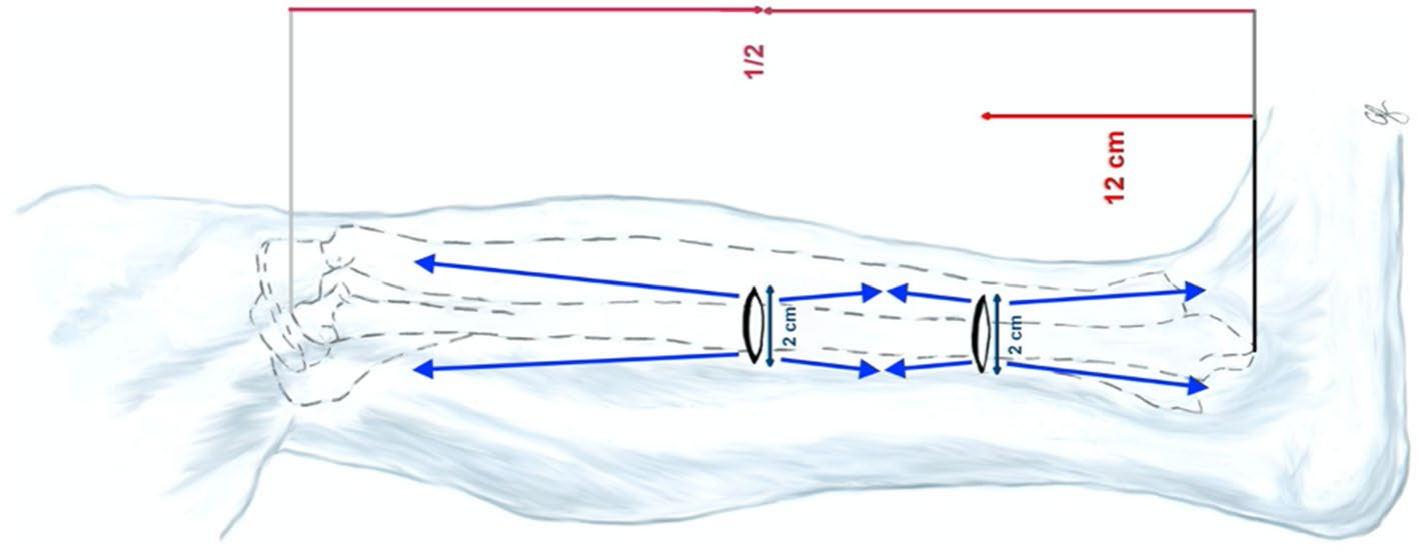
Double incision with fascia opening for decompression of the anterior and peroneal compartments with black arrows showing the long scissors position to perform the fascial incisions.

The incision over the anterior compartment was placed 1 cm medial to the SPN and was directed proximally to the tibial tuberosity and distally to the anterolateral edge of the tibia facing their landmarks. The incision over the peroneal compartment was placed 1 cm lateral to the SPN and directed towards their landmarks proximally to the fibula head and distally to the lateral malleolus (Fig. 1).

The mid-point of the fibula (half-way between the fibular head and the lateral malleolus) was identified and marked. A 2 cm horizontal skin incision was performed, followed by blunt subcutaneous dissection down to the fascia using dissecting scissors. The opening of the compartment coming from the distal side was continued proximally to the landmarks (Fig. 1).

#### Compliance with ethical standards

All investigated cadavers were donated to the Division of Macroscopic and Clinical Anatomy of the Medical University Graz under the approval of the Anatomical Donation Program of the Medical University of Graz and according to the Austrian law for donations.

### Statistical analysis

All evaluated data were exported to Microsoft Excel spreadsheets (Microsoft Excel 2010; Microsoft, Redmond, WA, USA) for descriptive statistical analysis. Continuous variables are presented as mean and standard deviation (SD), minimum and maximum, categorical data as frequencies and percentages.

## Results

### Fasciotomy of the anterior and peroneal compartments

A total of 40 lower extremities, 20 left-sided and 20 right-sided, were used for fasciotomy of the anterior and peroneal compartments using two horizontal fascial incision, one at the basic reference point and one over the mid-point of the Fibula. Complete release of the anterior compartment and peroneal compartment occurred in all specimens.

**Figure 2:**
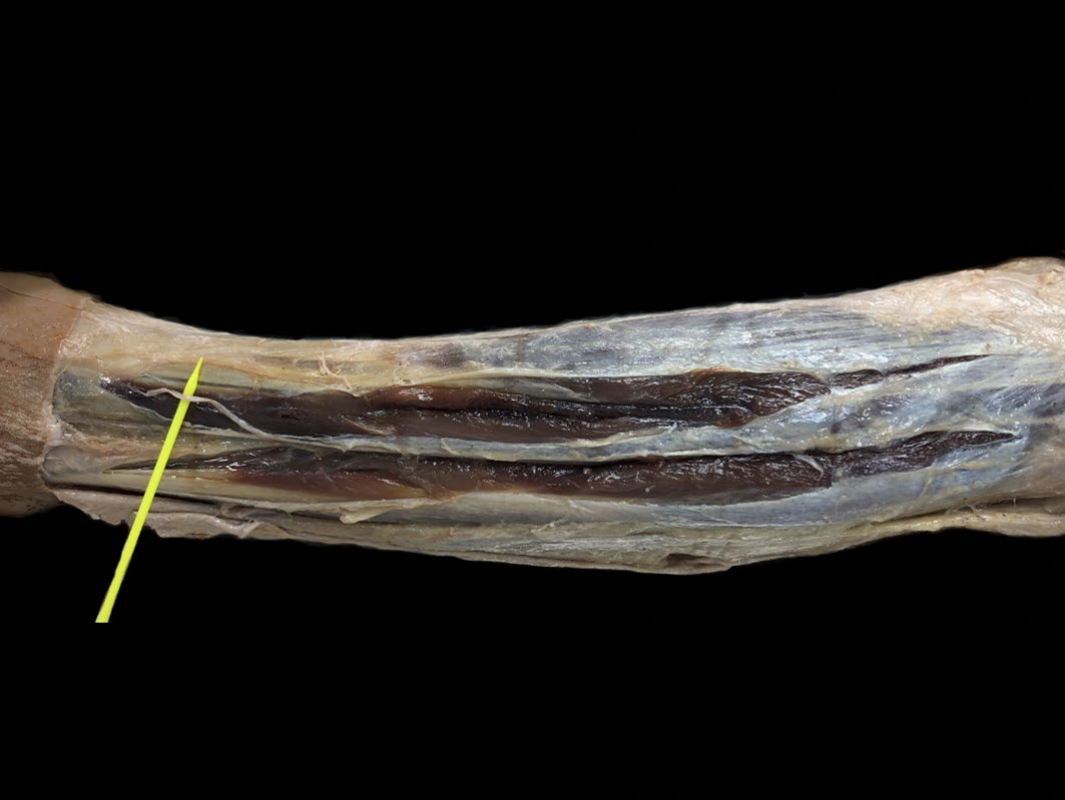
Dissected cadaveric specimen with fasciotomy of the anterior and peroneal compartments. The injured medial dorsal cutaneous nerve is identified over the yellow marker.

Forty anterior compartments underwent this approach. In all cases the fascia was completely divided. The mean length of the fascial division was 23.4 cm (SD: 2.1). Complete release of the anterior compartment occurred in all specimens, with nerve injury being identified in 2 specimens. More precisely, in these cases the medial dorsal cutaneous nerve got injured during the fascial opening of the anterior compartment (Fig. 2). It must be noted that these two nerve injuries happened during the preparation by the students.

Additionally, 40 peroneal compartments underwent this approach. In all cases the fascia was completely divided. The mean fascial opening of the peroneal loge was 21.7 cm (SD: 2.6) and nerve injuries occurred in none of the specimens.

**Figure 3:**
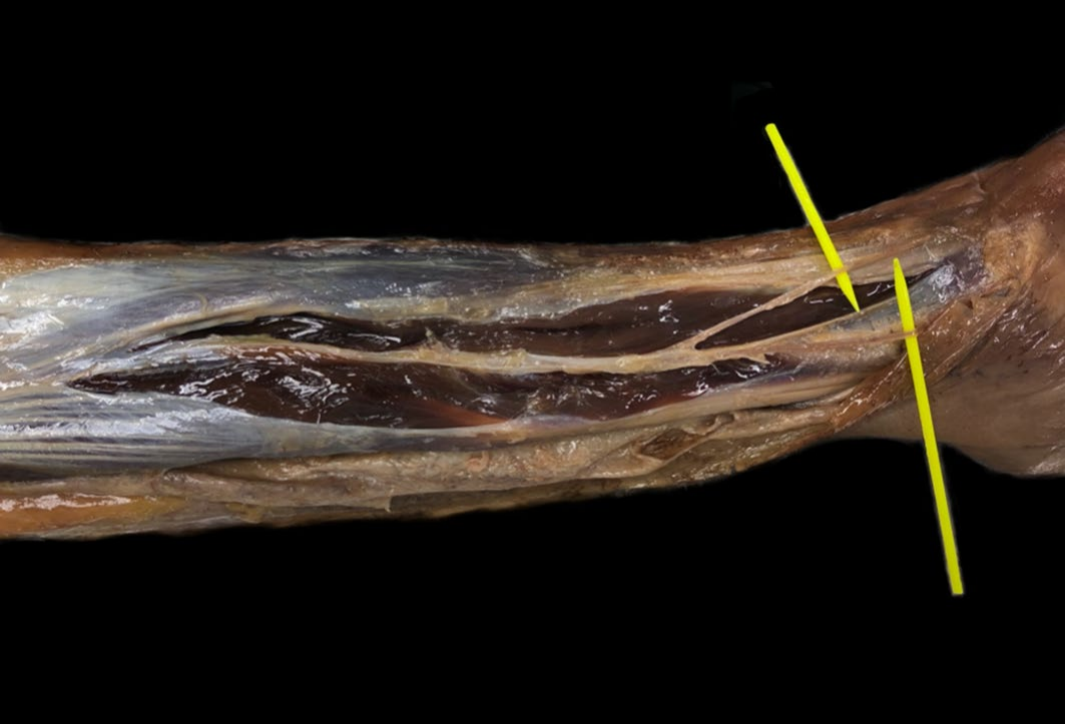
Dissected cadaveric specimen with fasciotomy of the anterior and peroneal compartments. The superficial peroneal nerve (SPN) is identified over the yellow markers.

The level at which the SPN pierces the superficial fascia of the leg and penetrates into subcutaneous cellular tissue is important. On average, the SPN becomes superficial at 11.6 cm (SD: 1.4) proximal to the lateral malleolus (Fig. 3).

Furthermore, the distances of the respective fascial openings at the most proximal and distal points to each other and to the edge of the tibia were measured, to control a continuous and consistent fascial opening. The anterior compartment showed an average of 2.5 cm proximal and 1.9 cm distal to the tibial edge. The peroneal compartment showed an average of 4,1 cm proximally and 3.3 cm distally to the tibial edge. The compartments to each other resulted in an average of 2.1 cm proximally and 1.9 cm distally. For a detailed overview regarding results, see Table 1.

**Table 1:** Details of fasciotomy outcomes.

Laterality	Right		Left	
Compartment incised	Anterior (n = 20)	Peroneal (n = 20)	Anterior (n = 20)	Peroneal (n = 20)
*Outcome*				
Complete division of fascia, n (%)	20 (100)	20 (100)	20 (100)	20 (100)
Mean length of fascial incision in cm (SD)	23.44 (2.1)	21.51 (3.4)	23.37 (2.1)	21,83 (1.5)
Muscle damage, n (%)	1 (5)	0 (0)	0 (0)	1 (5)
Nerve injury, n (%)	0 (0)	0 (0)	2 (10)	0 (0)
SPN tread out proximal to malleolus lateralis in cm (SD)	11.88 (1.4)		11.25 (1.3)	

## Discussion

After trauma of the lower extremity with suspected compartment syndrome it is necessary to open all four compartments, which can be done from a lateral approach on one side or by double incision on the medial and lateral sides. This can also be performed as an open procedure under direct view to verify the intactness of the muscles^14^.

Possible fasciotomy techniques have been under discussion in various articles recently. This cadaveric study demonstrated that a minimally invasive two incision approach to decompress the anterior and peroneal compartments of the lower leg is safe. As the study clearly shows, initial exploration of the SPN is a safe option for performing a fasciotomy in chronic compartment syndrome.

To our knowledge, this is the first study in the literature demonstrating that two incision minimally invasive fasciotomy can be used to safely decompress the anterior and peroneal compartment.

De Bruijn et al.^10^ investigated the safety of a modified percutaneous fasciotomy technique to decompress the anterior compartment using 9 cadaveric legs and 64 patients. Despite small numbers, they found no iatrogenic neurovascular injuries. A larger study on 118 patients also demonstrated a low rate (2%) of iatrogenic neurovascular injury^7^. In our study 2 (5%) nerval injuries occurred. Therefore, the results are comparable to the abovementioned studies, confirming the safety of a minimally invasive approach to the anterior compartment.

In patients with chronic functional compartment syndrome, incipient compartment syndrome or undergoing prophylactic fasciotomy, a minimally invasive technique would be indicated due to cosmetic outcomes and reduced soft tissue trauma. Prophylactic, adequate and early fasciotomy should be considered by the vascular surgeon in every case of vascular trauma when the ischemic time is greater than 6 h^15,16^. Should the need arise intraoperatively, the minimally invasive technique can be extended to the open invasive technique at any time without disadvantages for the patient.

Papalambros et al.^16^ concluded that endoscopically assisted fasciotomy has a lower risk of neurovascular injury when compared to a percutaneous technique in a cadaver study^16^. However, this study detected a surprisingly large number of nerve injuries including complete transection of the SPN and of the saphenous nerve in 4 out of 6 specimens. Certain differences in surgical technique may explain this disparity. Hutchinson et al.^11^ used considerably longer average single fasciotomy incisions compared to those in our study (30.1 cm for the anterior and 28.8 cm for the peroneal compartments). However, studies have demonstrated good clinical results using fasciotomy incisions as small as 4 cm in the anterior compartment^4,17^.

In our study a nerve injury is defined as a visible lesion caused by a discontinuity of individual nerve fibres or by a contusion of the nerve during preparation by scissors. The most important part of a nerve injury is the patient’s functional status and symptoms which can be evaluated clinically.

As a limitation of this study, fasciotomy was solely performed in a cadaveric sample. Therefore, it stays questionable whether this technique allows sufficient decompression of an acute compartment syndrome of the lower leg. With an open fasciotomy, hemostasis is achieved directly, whereas with a percutaneous technique a possible injury to subcutaneous/perforating veins could be possible (especially in patients with varicosis or postthrombotic syndrome. Furthermore, this is a cadaveric study, in the daily clinical or surgical practice it could be useful to perform a follow-up examination to evaluate other potential technical problems (e.g. muscle herniation risk).

## Conclusion

Two incision minimally invasive fasciotomy to decompress the anterior and peroneal compartments of the lower leg appears to be safe with regard to the results of this study. The key is to start the incision of the fascia in a safe distance to the SPN where it pierces the superficial fascia in the lower leg and courses into the subcutaneous tissue. An advantage when compared to other methods is that from the basic reference point, where the SPN becomes subcutaneous, the incision is made from proximal to distal, thus sparing the nerve. This new technique also offers the possibility for primary skin closure of two small skin incisions with cosmetic and functional benefits compared to other techniques. However the clinical indications for different fasciotomy techniques vary, a minimally invasive technique as described in this study would likely only appropriate for elective chronic exertional compartment syndrome fasciotomy, rather than acute compartment syndrome. Although this new technique seems to be a safe technique for fasciotomy of the anterior and peroneal compartments, especially for CECS, in an anatomical setting further clinical investigation of this technique needs to be done.
